# Knowledge and Perception of Community Based Integrated Management of Neonatal and Childhood Illnesses among Medical Students at a Medical College: A Descriptive Cross-sectional Study

**DOI:** 10.31729/jnma.8637

**Published:** 2024-07-31

**Authors:** Swasti Bhandari, Praneesh Ghimire, Tenzin Lhamo Lama, Lok Raj Joshi, Samata Nepal

**Affiliations:** 1 Lumbini Medical College and Teaching Hospital, Palpa, Nepal; 2 Department of Clinical Physiology and Biophysics, Karnali Academy of Health Sciences, Jumla, Nepal; 3 Department of Community Medicine, Lumbini Medical College and Teaching Hospital, Palpa, Nepal

**Keywords:** *children*, *illness*, *integrated management*, *medical students*, *neonate*

## Abstract

**Introduction:**

Community-Based Integrated Management of Neonatal and Childhood Illnesses (CB-IMNCI) is the integrated approach for the management of children's common health concerns in outpatient primary health care settings and interventions at the family and community level. This study aimed to assess the knowledge and perception regarding CB-IMNCI in medical students studying in the clinical phase of a medical college.

**Methods:**

A descriptive cross-sectional study was conducted from February to June 2023 among 218 clinical-year medical students after obtaining ethical clearance from the Institutional Review Committee (Reference number: IRC-LMC-04/M-022). A self-administered questionnaire with CB-IMNCI-related multiple-choice questions was used for data collection and the responses to knowledge-related questions were evaluated using a predefined answer key. The results were expressed in terms of the number and percentage of the participants who answered each questions correctly.

**Results:**

Of the 218 students, 111 (50.92%) were male and 107 (49.08%) were female. Among the participants, 164 (75.23%) (70-80% at 95% Confidence Interval) demonstrated basic knowledge of CB-IMNCI, successfully answering 50% or more of the questions. Among the male, 81 (72.97%), and among the female, 83 (77.57%) had basic knowledge of CBIMNCI. In terms of semester-wise distribution, 33(53.22%) in the 5^th^ semester, 43 (82.69%) in the 7^th^ semester, 41 (80.39%) in the 9^th^ semester and 47 (88.67%) were able to answer 50% or more of the questions correctly.

**Conclusions:**

This study showed that one fourth of the students lack the basic knowledge about CB-IMNCI. It suggests the need for further work to enhance effectiveness of pre-service CB-IMNCI training.

## INTRODUCTION

In 2015, the Ministry of Health and Population combined Community-based newborn care program (CB-NCP) and Community-based integrated management ofchildhood illness (CB-IMCI) to form CB-IMNCI.^1,2^ It deals with diseases like pneumonia, diarrhea, malaria, measles, and malnutrition in children between two months up to five years as well as the primary issues of newborns, like birth asphyxia, bacterial infection, jaundice, hypothermia, and low birth weight.^3-6^

Most childhood mortality can be prevented with effective interventions and quality care with the best use of the available resources.^7,8^ Hence, there is a need for proper knowledge and a review system of the CB-IMNCI strategy.^9^

CB-IMNCI content is now part of MBBS curricula in Nepal.^10^ A study in Karachi found that 80.4% of students answered over 50% of related questions.^11^ However, knowledge and perception assessment remains pending. Such assessments can guide improvements in pre-service training. ^6^

Our study aimed to assess the knowledge and perception regarding CB-IMNCI among the medical students of Lumbini Medical College and Teaching, hospital, Palpa Nepal.

## METHODS

A descriptive cross-sectional study was conducted in Lumbini Medical College and Teaching Hospital, Palpa from February 2023 to June 2023 after obtaining ethical clearance from the Institutional Review Committee with Reference number: IRC-LMC-04/M-022. Informed consent was obtained from all participants before the collection of the data.

The study intended to include students of Lumbini Medical College enrolled in the clinical phase and the internship. As per the curriculum of Kathmandu University, the MBBS program comprises the preclinical phase referring to the first two years, and the clinical phase referring to the next two and half years. One year of compulsory internship follows the clinical phase. In terms of the semesters, the first two years correspond to the first to fourth semesters and the next two and half years correspond to the fifth to ninth semesters. We excluded students who did not consent to the study.

Non-probability sampling using the convenience sampling method was applied and the sample size was calculated.

The sample size was calculated using the formula:


$$\begin{array}{ll} \text{n} & ={\text{Z}}^{2}\times \frac{\text{p}\times \text{q}}{{\text{e}}^{\text{2}}}\\ & =1.96^{2}\times \frac{0.5\times 0.5}{0.05^{2}}\\ & =385 \end{array}$$

Where,

n = minimum required sample size
Z = 1.96 at 95% Confidence interval (CI)
p = 0.5 (Taken for maximum sample size calculation)
q = 1-p
e = margin of error, 5%

The calculated sample size was 385.

The total population of students was N=400, sample size for finite population was calculated


$$\begin{array}{ll} \text{n} & =\frac{\text{n}}{1+\frac{\text{n}-1}{\text{N}}}\\ \text{n} & =\frac{\text{385}}{1+\frac{\text{285}-1}{\text{400}}}\\ & =197 \end{array}$$

The calculated minimum required sample size was 197.

15% non response rate was added: n = n + 15% of n = 227

A self-administered multiple choice questionnaire (provided as the supplementary material) was adapted from a previous similar study conducted among the final year medical students in Karachi, Pakistan^11^ and the IMCI preservice education guide by WHO.^6^ The research team, including the faculty member from the Department of Community Medicine, adapted and finalized the questions and prepared the answer key. The questionnaire was also pretested before data collection. A few questions contained multiple correct alternatives. There were eight questions to assess perception and twelve questions to evaluate the knowledge of CB-IMNCI.

Questionnaires were distributed to the participants in respective classrooms, clinical posting classes and hostel rooms. Each of the participants was asked to return the completed questionnaires on the next day and was followed up to collect the same.

Data was compiled and analyzed using Microsoft Excel 2019 and R packages 'tidy verse' (version 1.3.1) in the statistical software environment R (version R-4.1.1). Response to each question regarding knowledge was evaluated according to the answer key. In case of multiple correct alternatives, only the responses that selected all of the correct alternatives were marked correct. Those students who were able to answer 50% or more of the questions correctly, were considered to have the basic knowledge on CBIMNCI. The results were expressed in terms of the number and percentage of the participants who answered each question correctly.

## RESULTS

Out of the 227 questionnaires distributed, 218 (94.78%) were collected from the participants. Male participants were 111 (50.92%). Among all the participants, there were 53 (24.31%) interns, 51 (23.39%) students were in the 9th semester, 52 (23.85%) students in the 7^th^ semester, and 62 (28.44%) students in the 5th semester at the time of data collection.

Out of all the participants, 164 (75.23%), demonstrated basic knowledge of CB-IMNCI by correctly answering 50% or more of the questions. Specifically, 81(72.97%) male and 83 (77.57%) female had basic knowledge of CBIMNCI.

**Figure 1:**
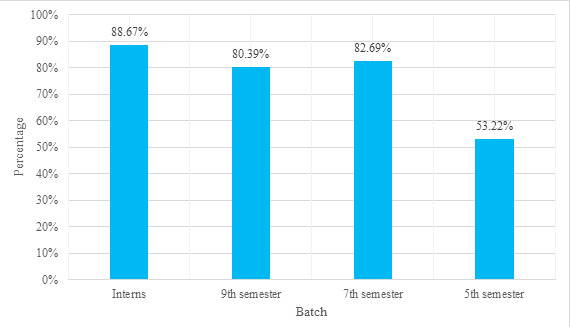
Percentage of participants in various batches having basic knowledge of CB-IMNCI (answering 50% or more question correctly) (n=218).

When examining the distribution by semester, 33 (53.22%) students in the 5^th^ semester, 43 (82.69%) students in the 7^th^ semester, and 41 (80.39%) students in the 9^th^ semester, as well as 47 (88.67%) interns, were able to answer 50% or more of the questions correctly (Figure 1).

Textbook was cited as a source of knowledge by 198 (90.82%) participants, and community medicine was the subject in which CBIMNCI was studied, as cited by 65 (29.81%) participants (Table 1).

**Table 1:** Source of knowledge and subject in which CBIMNCI was studied (n= 218).

Variable	n (%)
Source of knowledge of CBIMNCI^*^	
Textbook	198 (90.83)
Classroom	187 (85.78)
Clinical posting	130 (59.63)
Government/WHO Publication	75 (34.40)
Friends	73 (33.49)
Subject in which CBIMNCI was studied	
Community Medicine	65 (29.82)
Pediatrics	2 (0.92)
Both	151 (69.27)
None	-

* Each individual could choose multiple sources.

Upon response of the participants towards perception questionnaire, 216 (99.08) of the participants stated that CBMINCI approach is important and 208 (95.41) participants stated that it reduces childhood mortality (Table 2).

**Table 2:** Responses to the questions about perception towards CB-IMNCI (n = 218).

SN Questions	Yes	No	Not sure
1. Are CBIMNCI guidelines being practiced regularly?	150 (68.81)	68 (31.19)	-
2. Do you think CBIMNCI approach adds anything important?	216 (99.08)	2 (0.92)	-
3. Do you think CBIMNCI reduces childhood mortality/morbidity?	208 (95.41)	-	10 (4.59)
4. Was the lecture on CBIMNCI helpful?	204 (93.58)	14 (6.42)	-
5. Do you think CBIMNCI will help you in clinical practice?	207 (94.95)	-	11 (5.05)

* Option was not present in that question

Upon response to the knowledge questionnaire, the correct responses ranged between 211 (96.78%) a 24 (11.01%) (Table 3).

**Table 3:** Responses to the questions to test knowledge about CB-IMNCI content (n = 218).

SN.	Questions	n (%)
1.	What are the diseases/health issues included in CB-IMNCI guidelines?	177 (81.19)
2.	What is age included in CB-IMNCI guideline?	151 (69.26)
3.	In CB-IMNCI Chart which color coding indicates the referral to higher center?	211 (96.78)
4.	What are the conditions that should be assessed in an infant up to two months?	-
5.	Arrange the components of assessment of a sick child aged up to two months in correct order as stated in the CB-IMNCI guideline.	54 (24.77)
6.	Which of the following are criteria for good attachment to the breast?	60 (27.52)
7.	Which of the following conditions does Kangaroo mother care (mayako angalo) prevent or correct?	206 (94.49)
8.	What are the danger signs/symptoms to be checked for in children aged two months up to five years?	120 (55.04)
9.	What are the conditions that should be checked for in children between two months and five years?	206 (94.49)
10.	What are the nutritional deficiencies taken into consideration in children between two months and five years?	93 (42.66)
11.	What are the points to be included for the counselling of the mother of a sick child in the home treatment for diarrhea?	24 (11.01)
12.	What is the cut off value for fast breathing in a child who is 10-month-old?	24 (11.01)

## DISCUSSION

This study aimed to assess and evaluate the awareness of integrated management of neonatal and childhood illnesses among medical students.

This study revealed that 164 (75.23%) of the students had basic knowledge regarding CB-IMNCI, which was predefined as the ability to answer 50% or more questions in the questionnaire given. This result indicates room for improvement as about a quarter of the respondents were unable to answer 50% of the questions given. A parallel study conducted among final-year medical students in Karachi, Pakistan uncovered that 80.4% of the students were capable of answering 50% or more of the questions in the provided questionnaire. So, their performance in this regard was superior to that of our study.^11^ However, some of the questions in the questionnaire differed between the studies.

Regarding the source of information about CB-IMNCI most of the students selected 'Textbook' followed by 'classroom' and 'clinical posting' as their source. Use of government publications as the source of knowledge was relatively low possibly due to lack of interactive programs between the government and the medical college. In the context of the subject in which CB-IMNCI was discussed, most of the participants (above two-thirds) chose both community medicine and pediatrics although about one-third chose community medicine only.

Regarding the questions about perception towards CB-IMNCI, most of the participants i.e., 150 (68.81%) agreed that CB-IMNCI is being practiced regularly while 68 (31.19%) thought otherwise. This shows the need to practice CB-IMNCI frequently for proper implementation. The findings from the study indicated that 216 (99.08%) of the participants were in agreement that the CB-IMNCI approach adds something important while 2 (0.92%) disagreed. When participants were asked whether they believed CB-IMNCI reduces childhood mortality/morbidity, the majority 208 (95.41%) agreed, demonstrating strong support for its effectiveness. Conversely, a small percentage 10 (4.59%) expressed uncertainty, indicating that they were not sure about the effectiveness of CB-IMNCI in reducing childhood mortality/morbidity. The majority of participants, comprising 204 (93.58%), found the lectures on CB-IMNCI to be helpful while a minority of 14 (6.42%) did not agree with its usefulness. Additionally, 207 (94.95%) of students believed that CB-IMNCI would assist them in their clinical practice, highlighting its perceived value. In contrast, 11 (5.05%) of students were uncertain about its usefulness in clinical practice.

When participants were asked to identify the diseases/health issues included in CB-IMNCI guidelines through a multiple-choice question, the results revealed that 177 (81.19%) participants answered all questions correctly. A significant number of 210 (96.33%) respondents correctly identifieddiarrhea. Pneumonia closely followed with 204 (93.57%) votes, while measles, sudden infant death syndrome, and road traffic accidents received 201 (92.20%), 14 (6.42%), and 8 (3.66%) responses, respectively. The similar study in Karachi, Pakistan reveals that out of the total 184 who answered, no student from the private universities answered all the options correctly while 176 (95.5%) answered one out of two options correctly.^11^ Also the overall consistency of CB-IMNCI management seems to be poor in the various studies conducted on this topic.^12-15^

The findings indicate that 151 (69.26%) of participants correctly identified the age group of children included in CBIMNCI as birth up to 5 years. This suggests that a significant proportion lacked adequate knowledge or were unsure about the age range. Regarding the color-coding indicating referral to a higher center yielded a highly favorable result, 211 (96.78%) of participants correctly identified the color red as the indicator for referral, while the remaining participants chose other options. A mere 54 (24.77%) of participants were able to correctly identify the conditions that should be assessed in an infant up to two months as umbilical infection, low birth weight, and jaundice. This suggests that the majorityof participants lacked properknowledge or understanding of these conditions, highlighting a gap in their awareness. Approximately 120 (55.04%) of participants successfully identified the correct criteria for good attachment of the breast, which was indicated by the chin touching the breast and the mouth being wide open. This suggests that a little over half of the participants accurately recognized the key elements of proper attachment during breastfeeding. A remarkable 206 (94.49%) of participants were able to correctly identify hypothermia as a condition prevented by kangaroo mother care. It seems that the students had a good knowledge of this topic.

When participants were asked about the danger signs/symptoms to be checked for in children aged two months up to five years, the results revealed that 158 (72.48%) of participants correctly identified convulsions and vomiting as the correct options. Additionally, 141 (64.68%) of participants selected unconsciousness as a danger sign. However, 64 (29.36%) of participants chose cyanosis, which was not the correct option as per the CB-IMNCI guideline. Notably, all three correct options were answered accurately by 78 (35.77%) of the participants. There were two questions that were answered correctly by very few students i.e. 20 (9.17%)of the students correctly answered the question which asked about conditions that should be checked for in children between two months and five years and the same proportion answered the question on the nutritional deficiencies taken into consideration in children between two months and five years.

This type of research study has not been done before in Nepal and this study depicts the picture regarding the knowledge and perception of the students about CB-IMNCI while they are still in the medical college before entering their independent job responsibility. Strengthening of the learning in this phase may be expected to enhance its better implementation in the on the job health care settings. In the future, it may help tackle inadequate implementation of CB-IMNCI Program at Primary Health Care Centers and Health Posts.^16,17^

This study has some limitations. Convenience sampling was conducted due to geographical proximity, availability of the participants at the given time, or willingness to participate in the research. So it carries the risk of introducing sampling bias although we were not aware of any systematic differences between the students who participated in the study and those who did not. Further, a questionnaire with a greater number of questions would be able to cover a wider content area.

## CONCLUSIONS

Our study showed that one fourth of the students lack the basic knowledge about Community-Based Integrated Management of Neonatal and Childhood Illness (CB-IMNCI). It indicates the need for more radical and practical approach to pre-service education on CB-IMNCI. We highlight the necessity for enhancing our current curriculum and pedagogical approach.
